# Perspective on Global Measles Epidemiology and Control and the Role of Novel Vaccination Strategies

**DOI:** 10.3390/v9010011

**Published:** 2017-01-19

**Authors:** Melissa M. Coughlin, Andrew S. Beck, Bettina Bankamp, Paul A. Rota

**Affiliations:** Measles, Mumps, Rubella, and Herpesvirus Laboratory Branch, Division of Viral Diseases, Centers for Disease Control and Prevention, 1600 Clifton Rd., Atlanta, GA 30329, USA; mcoughlin@cdc.gov (M.M.C.); abeck@cdc.gov (A.S.B.); bbankamp@cdc.gov (B.B.)

**Keywords:** measles, elimination, surveillance, vaccine, novel vaccination strategies

## Abstract

Measles is a highly contagious, vaccine preventable disease. Measles results in a systemic illness which causes profound immunosuppression often leading to severe complications. In 2010, the World Health Assembly declared that measles can and should be eradicated. Measles has been eliminated in the Region of the Americas, and the remaining five regions of the World Health Organization (WHO) have adopted measles elimination goals. Significant progress has been made through increased global coverage of first and second doses of measles-containing vaccine, leading to a decrease in global incidence of measles, and through improved case based surveillance supported by the WHO Global Measles and Rubella Laboratory Network. Improved vaccine delivery methods will likely play an important role in achieving measles elimination goals as these delivery methods circumvent many of the logistic issues associated with subcutaneous injection. This review highlights the status of global measles epidemiology, novel measles vaccination strategies, and describes the pathway toward measles elimination.

## 1. Introduction

Measles virus (MeV) is the etiologic agent of measles, a highly contagious and vaccine-preventable illness. Despite the existence of a safe and effective vaccine, measles remains a major cause of morbidity and mortality globally, especially in young children. This review focuses on the global measles burden and the contribution of current and novel vaccination strategies to reaching the World Health Organization (WHO) measles elimination goals. Relevant background information on the virus and the disease is included to facilitate discussion.

MeV is a member of the family *Paramyxoviridae*, genus *Morbillivirus* and has a negative-sense, single-stranded RNA genome measuring approximately 15.9 kb [1]. The MeV particle is pleomorphic and is largely cell-associated with a virion size ranging from 50 to 510 nm [1,2]. In the virus particle, two surface glycoproteins, the fusion (F) and hemagglutinin (H), form a multimeric complex which mediates viral entry. The nucleoprotein (N) encapsidates the viral genome and is the most abundant viral protein in the virion and in infected cells [1].

Entry to the host cell is initiated by binding of H to one of three host cell receptors, CD150 (signaling lymphocyte activation marker or SLAM), nectin-4 or CD46, followed by membrane fusion facilitated by F. CD150 is expressed on many cell types of the immune system including dendritic cells, lymphocytes and some macrophages [3]. Nectin-4 is expressed basolaterally at the lymphocyte-accessible adherens junctions of the respiratory epithelium [4,5,6]. The capacity to bind CD46, which is expressed on all nucleated human cells, is acquired by viruses passaged in cell culture and both vaccine and laboratory-adapted strains use CD46 as a receptor [7,8,9].

H, and to a lesser extent F, are targets for neutralizing antibodies. Despite the existence of multiple genotypes, MeV strains are serologically monotypic, and polyclonal antibody raised against the vaccine strains will neutralize infectivity of all other genotypes [10]. This property is attributed, in part, to limitations in generation of non-deleterious mutations within the H protein. Key residues conferring binding capacity to SLAM, the receptor for wild-type (WT) viruses, are highly conserved, and unable to tolerate changes that result in loss of neutralizing ability [10,11,12]. Monoclonal antibody escape mutants are still efficiently neutralized by serum from vaccinees, and serum from vaccinees neutralizes representative strains from currently circulating genotypes of WT MeV [10,12,13,14,15].

## 2. MeV Infection and Pathogenesis

MeV is highly infectious to humans, causing a self-limiting, febrile illness characterized by a maculopapular rash. MeV is transmitted by infectious aerosols. Studies in animal models indicate that the initial target cells are alveolar macrophages and dendritic cells which are infected via CD150 [16,17]. The virus is transported to draining lymphoid tissues, seeding a systemic infection with preferential tropism to B and T-lymphocytes. The incubation period is estimated to last 10 to 14 days, and is associated with leukopenia. The prodromal phase is marked by onset of fever accompanied by cough, coryza, and/or conjunctivitis. Rash is generally observed from three to five days following onset of fever. Viral shedding begins in the prodromal phase prior to rash onset. Following viremia mediated by infected lymphocytes, the respiratory epithelium is infected basolaterally via nectin-4 and viral transmission continues through respiratory secretions [4,5,6,18]. Individuals are considered to be infectious from approximately four days before rash to four days after rash onset. Total uncomplicated disease course is 17–21 days from first sign of fever [19].

Recovery from measles produces lifelong immunity; however, during and after acute infection, the patient paradoxically experiences transient immunosuppression, which is substantiated by the suppression of delayed-type hypersensitivity responses [20,21]. Due to MeV-induced immunosuppression, secondary bacterial infections frequently occur, causing pneumonias or gastrointestinal infections, which are the leading cause of morbidity and mortality associated with measles [22]. A recent modeling study suggested that increased susceptibility to infections may continue for 2–3 years after measles [23]. Secondary infections and the associated morbidity and mortality are prevented by measles vaccination.

Typically, measles is self-limiting; however, a number of severe outcomes have been described. The risk of measles morbidity and mortality is multifactorial, depending on both vaccination and nutrition status; malnutrition and vitamin A deficiency are risk factors for severe outcomes [24,25]. Infrequently, measles may result in complications involving the central nervous system (CNS) and several distinct pathologies have been described [26] including primary measles encephalitis (PME) [27], acute disseminated encephalomyelitis (ADEM) [28], measles inclusion body encephalitis (MIBE) [29], and subacute sclerosing panencephalitis (SSPE) [28,30]. SSPE is a rare, fatal neurological complication of measles caused by a persistent MeV infection with an estimated incidence of 1:9000 to 1:15,000 measles cases in the US [30]. SSPE is characterized by infection of the central nervous system, progressive neurological deterioration, and a protracted terminal disease course of 1–3 years. SSPE is a complication of infection with WT MeV that occurs on average 7–10 years following the initial MeV infection, and is prevented by vaccination [28,30,31]. Vaccine strains have not been detected in SSPE [30].

## 3. Epidemiology

In the pre-vaccine era, outbreaks occurred in late winter and early spring in temperate climates, whereas in tropical climates outbreaks mainly occurred in the dry season with continual low-level measles transmission year-round [32]. This seasonal nature of measles reflects high levels of contact clustering or population density; in developed countries, this corresponds to the public school calendar, and in tropical climates this corresponds to yearly migration of agricultural workers [33,34,35]. The size of the outbreaks varies with a periodicity of 2–5 years, during which susceptible individuals from multiple birth-cohorts accumulate in sufficient numbers to sustain large outbreaks. In the absence of vaccination, measles is a disease of young children [36]. Improved vaccination coverage reduces the likelihood of childhood exposure at earlier ages, exposing gaps in immunity and shifting the average age of infection. Under these conditions, all-ages incidence is reduced, however the relative proportion of cases is increased for both older age groups, where immunization gaps may have occurred and children under one year of age who are below the recommended age for vaccination [37,38,39,40].

The range of the basic reproduction number *R_0_* (a metric for transmissibility) for measles was estimated to be 9–18, exceeding that of other viral diseases (e.g., smallpox *R*_0_ = 5–7 and polio *R*_0_ = 4–13) [41,42]. Based on the high *R*_0_, the level of population immunity (herd immunity) necessary to interrupt measles transmission is approximately 95% [42]. Measles is often the first vaccine preventable disease detected following a breakdown in routine immunization activities, and as such is a “canary in the coal mine”, and a leading indicator of the status of vaccination programs [43].

The estimated global measles case burden exceeded 9.7 million cases in 2015 with 254,928 reported cases across all six regions of the WHO, with an estimated total of 134,200 (95% CI = 74,400–353,600) measles deaths [44]. One known limitation in measles case surveillance is that it is subject to underreporting. Studies have demonstrated underreporting in outbreak settings; one study in Germany demonstrated an average of three times lower rates of reporting to the mandatory notification system compared to health insurance claim submissions by doctors [45,46]. The Measles Strategic Planning (MSP) tool developed by the WHO for the analysis of immunization and surveillance data at a national level, also demonstrates underreporting in non-outbreak settings in countries across the globe [47]. The MSP tool also allows estimation of measles mortality based on the number and age of reported cases, measles containing vaccine (MCV) coverage and country specific case fatality ratios (CFR) [47,48,49]. Estimates of measles case fatality rates from 102 studies conducted between 1980 and 2008 indicated that the CFRs ranged from 0% to 40.16%, with a set of nationally representative studies showing a median CFR of 1.0%. Estimates of CFR varied widely between geographic region and subnational locality of study and were inversely correlated with economic development. In developed countries, CFRs were determined to be approximately 0.05%, but CFRs have reached 6% in developing countries among children aged 1–4 years [48]. Greater measles CFRs are observed in children during major humanitarian crises and are inversely correlated with access to vaccination [48]. The current global incidence of measles is 36 cases/million population, down from 146 cases/million population in 2000 [44,50]. Measles incidence estimates declined by 75% compared to 2000, and in 2015, 109 countries achieved an incidence of less than 5 cases/million population [44,50,51].

**Figure 1:**
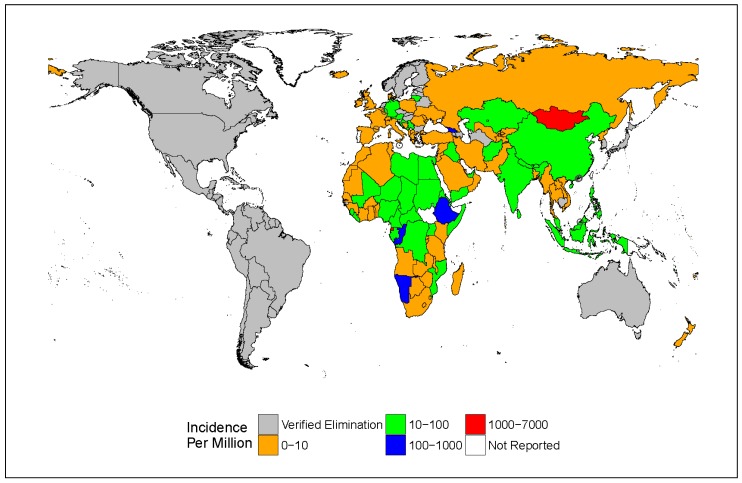
Current measles incidence and countries with verified elimination. Depiction of 2015 per-country measles incidence per million population, overlaid with countries for which measles elimination has been officially verified (grey) [53,54,55,56]. Grey circles indicate countries not readily viewable on the map. Verified elimination was obtained from documentation produced by the World Health Organization (WHO) regional elimination verification commissions [53,54,55]. Shapefile was obtained from NaturalEarth.com, 10-meter resolution, version 3.1.0, and was produced in R version 3.2.3.

Incidence varied by WHO region; the African, Eastern Mediterranean and European Regions reported increased incidence in 2014–2015 due to large measles outbreaks. The numbers of reported cases were 98,621 (incidence of 100/million) for the African region, 423 (0.6/million) for the region of the Americas, 21,335 (33/million) for the Eastern Mediterranean region, 25,974 (31/million) for the European Region, 29,109 (17/million) for the South-East Asian region and 65,176 (35/million) for the Western Pacific Region [44] (Figure 1). The region of the Americas verified the elimination of measles in 2016, demonstrating the feasibility of elimination in low- and middle-income countries [52,53].

## 4. Surveillance

Depending on geography and seasonality, the clinical presentation of measles may mimic those of other viral diseases. Furthermore, MeV infection of immunocompromised individuals may not result in typical measles symptoms, though often these individuals suffer from severe disease outcomes, particularly the development of pneumonia [57,58,59]. Therefore, the current measles elimination goals in all WHO regions necessitate case-based surveillance including laboratory confirmation of suspect cases [32]. With coordination from the WHO, the Global Measles and Rubella Laboratory Network (GMRLN) performs case-based laboratory surveillance using standardized methods [60]. The GMRLN has expanded greatly since its establishment in 2000; currently there are 703 laboratories located in 180 countries in all WHO regions. The network is made up of subnational and national laboratories, regional reference laboratories, and three global specialized laboratories.

Suspected cases that meet the WHO case definition for measles, which requires a finding of acute febrile disease (≥38.3 °C) featuring a generalized maculopapular rash lasting three days or longer, and coryza, cough, or conjunctivitis are classified in four groups. A *suspected* case meets the case definition with attendant suspicion of measles by the examining clinician. A *laboratory-confirmed* case meets these clinical criteria, with laboratory confirmation of infection, typically by detection of MeV-specific IgM antibodies in serum or by detection of viral RNA by reverse transcription (RT)-PCR. An *epidemiologically-linked* case meets the clinical criteria and has an epidemiologic link to a laboratory-confirmed case. A *clinically-compatible* case meets the clinical criteria in the absence of laboratory specimen collection.

Molecular surveillance in combination with standard epidemiologic information can be used to trace pathways of transmission and to provide evidence for verification of measles elimination. Sequence information can also distinguish between WT infection and vaccine-related rash. Genotypic surveillance of MeV is based on the nucleotide sequence encoding the 150 carboxyl-terminal amino acids of the N protein (N450), a region of high variability in the genome [61]. This method effectively differentiates between the 24 MeV genotypes, six of which (B3, D4, D8, D9, G3, and H1) currently remain in circulation worldwide [41,44,62,63]. At this writing, more than 33,210 sequences of MeV have been submitted from all WHO regions to the global Measles Nucleotide Surveillance (MeaNS) database [62]. Most of the sequences in MeaNS are N450, but submission of *N* gene and *H* gene sequences is recommended for reference strains. Reference sequences for each MeV genotype are available; however, because many of these viruses were isolated more than 10 years ago, the sequences are often divergent from currently circulating strains. While the WHO reference strains may be used to accurately identify the genotype of a new MeV sequence, the use of “named strains” allows for more sensitive comparison of currently circulating strains. The sequences of “named strains” are from MeVs that have widespread circulation in multiple countries and have been circulating for at least two years. A listing of named strains for all circulating virus genotypes can be found in MeaNS [62].

**Figure 2:**
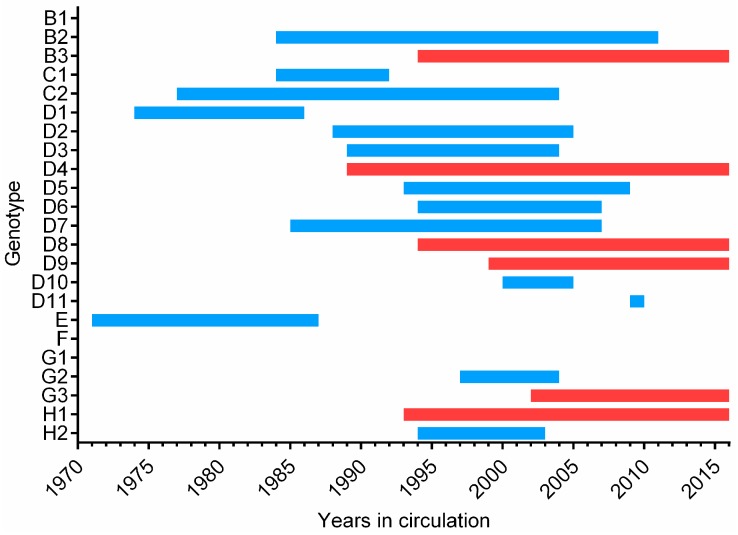
Year of first and last detection of Measles virus (MeV) wild-type genotypes. The first and last years of documented circulation reported to MeaNS for all 24 MeV genotypes is shown. First year detection determined from earliest reference strain reported to MeaNS and does not necessarily indicate first year of circulation. All viruses in red with a date of 2016 are currently circulating genotypes. Measles vaccine was derived from genotype A virus, which is not shown, and the earliest detection of genotype A was in 1954. Dates obtained from reference [62].

Data from virologic surveillance suggests a decrease in overall genetic diversity of measles (Figure 2), as the number genotypes in circulation has decreased [62]. Sequencing larger fragments of the MeV genome offers the potential for greater resolution of MeV transmission pathways, including differentiating between multiple sources of importation of the same genotype and co-circulating lineages in countries with endemic virus. Regions of interest for expanded sequencing include the noncoding region between the matrix (*M*) and *F* genes (MF-NCR) as well as the whole genome, which can be sequenced by Sanger or next-generation methods [64,65]. Information derived from the MF-NCR yields phylogenetic trees with similar topology to those obtained by whole genome sequencing, demonstrating that this region has utility for improving molecular surveillance of MeV [64,65,66].

Molecular testing carried out by the GMRLN is essential for measles case-based surveillance and to distinguish endemic from imported cases. Genotype information helps to characterize the elimination status of a country. Countries with endemic measles will show multiple co-circulating lineages of the endemic genotype(s). Countries that have eliminated endemic measles may find multiple genotypes representing different importation sources. Furthermore, these countries will not demonstrate sustained transmission of any lineage. Countries that have previously had high vaccination coverage but have begun to accumulate increased numbers of susceptible individuals may experience reintroduction of measles which can result in large outbreaks of a single viral lineage with identical or nearly identical sequences [11]. Molecular testing will be essential as countries approach elimination to provide laboratory evidence for the absence of endemic transmission of MeV.

Though detection of viral RNA by RT-PCR is playing an increasing role in case confirmation, immunoglobulin M (IgM) detection by enzyme immunoassay (EIA) is still the most commonly used diagnostic method. In addition, serological techniques play an important role in the evaluation of population immunity. Administrative coverage estimates are sometimes an inaccurate measurement of vaccination coverage at a population level [62]. Serosurveys have the potential to more reliably measure the levels of population immunity. Plaque reduction neutralization testing is the “gold” standard for evaluation of a protective antibody titer in serum; however, this method requires special laboratory skills and is highly labor intensive. The evaluation and use of new serological techniques such as the multiplex immunoassay (MIA) show promise to evaluate measles immunity within populations and will likely play a greater role in providing the measurements of population immunity required for verification of elimination [44,67].

## 5. Measles Vaccine

The first live attenuated measles vaccine was licensed in 1963. An early candidate vaccine, the Edmonston B vaccine strain, was derived by serial passage of the Edmonston strain in human and monkey kidney cells and chicken embryo fibroblasts but proved to be insufficiently attenuated [68]. A second, more attenuated generation of measles vaccines (Schwarz, Edmonston-Zagreb, AIK-C and Moraten strains) all belong to the Edmonston lineage and are currently used. Additionally, the three live-attenuated strains CAM-70, Leningrad-16, and Shanghai-191 were generated from non-Edmonston WT progenitors [32,68,69]. All measles vaccines are in genotype A. Sequences of measles vaccine strains do not differ greatly from each other; several conserved nucleotide changes between vaccine and WT strains have been documented and the functional consequences of these differences have been observed [70,71]. However, the molecular basis for attenuation is still unknown. Vaccination results in similar humoral and cellular immune responses as those generated by natural disease, though the antibody titer produced following vaccination is lower than that produced following natural infection [32,68].

At the same time, the live attenuated measles vaccine was developed in the 1960s a formalin-inactivated vaccine was also developed. However, this vaccine resulted in enhanced disease following exposure to WT virus, leading to atypical measles, in which vasculitis and pneumonitis were associated with immune complex formation and deposition in tissues [72]. Studies in non-human primates demonstrated that the immune response to the formalin-inactivated vaccine was characterized by the absence of cytotoxic T cells and by antibodies of low avidity due to the lack of affinity maturation. These antibodies waned quickly and could neutralize vaccine virus which used CD46 as a receptor but did not neutralize WT virus which used CD150 [68,72]. The inactivated vaccine was initially licensed in 1963, but withdrawn in 1967 [68].

Another vaccine preparation that was determined to be unacceptable due to adverse events was vaccination with high titer (equivalent to or greater than 40,000 plaque-forming units (pfu)), live-attenuated measles vaccine which was explored in the 1990s. Initial studies showed that use of high titer vaccine led to comparatively higher seroconversion rates in younger infants and could partially overcome maternally acquired antibodies, which hinder the development of an effective immune response. However, antibody levels in high titer vaccinated younger infants remained lower than those of infants vaccinated with standard dose at nine months [73,74]. The recommendation to use high titer vaccine was withdrawn by the WHO after studies revealed increased mortality within 2–3 years following measles vaccination, especially in young girls. Theories for the increased mortality rate in girls included increased immunosuppression by the high titer vaccine leaving children more susceptible to secondary infections, and the change in sequence of vaccine delivery [68,75,76,77]. Some studies have linked increased mortality in girls with receipt of an inactivated vaccine following measles vaccination [75,76,77].

The current MeV vaccine is safe and highly effective at providing protection against measles. The goal of the current vaccination schedule is to provide optimal vaccine efficacy, ideally by administration to an infant following decay of maternal antibody, and before the likelihood of the child being exposed to MeV [32,68]. Currently, the WHO recommends administration of the first dose of measles-containing vaccine (MCV) at nine months in measles-endemic regions and at 12–15 months in non-endemic regions [32,68]. During an outbreak of measles, the first dose may be given as early as six months to protect infants under one year of age, with a follow-up dose at 9–12 months [68,78]. After a single dose of MCV at nine months, 85% of children develop protective immunity to measles, and when the first dose is given at 12 months, 95% of children are protected from measles [32,68]. A two-dose vaccination strategy is recommended to achieve the 95% coverage within a community to provide sufficient herd immunity to interrupt transmission by this highly contagious pathogen. The second dose also targets vaccinees that did not achieve an adequate immune response following a single dose of MCV. When countries have sufficient health system infrastructure, the administration of the second dose of MCV may be achieved as part of routine immunization and school entry requirements [32,68]. In other countries, supplemental immunization activities (SIAs) are important to achieve high vaccination coverage and to immunize children that may have been missed during routine immunization or previous SIA activities [11,32,68].

## 6. Novel Vaccination Strategies

Despite the availability of an effective vaccine delivery system, several alternative vaccination strategies are under investigation. The motivations behind the desire to improve upon the current vaccine delivery method include eliminating or reducing the cold chain requirements, concerns regarding injection safety and contaminated waste disposal, and decreasing the need for medically trained personnel to administer the vaccine [68]. The current live attenuated measles vaccine rapidly loses potency once reconstituted. Within one hour of reconstitution if the vaccine is stored at 20 °C, it will lose approximately half of its titer; if stored at 37 °C, it will lose nearly all of the 10^3^ plaque forming units required for adequate vaccination [68]. An example of the result of cold chain breakdown was demonstrated in the Marshall Islands, in which secondary vaccine failures resulted from a compromised cold chain and lead to the first outbreak of measles in more than two decades [78]. Furthermore, alternative routes of immunization are being explored to determine if the inhibitory effects of maternal antibody may be overcome to allow for immunization of infants at a younger age, particularly in regions of high endemicity [32,68]. The alternative routes of vaccination currently under evaluation are vaccination via the respiratory route and the intradermal route by microneedles which are described below. Other alternative vaccination devices such as needle-free disposable cartridge jet injectors have also been evaluated with intent to eliminate the logistics of sharps usage as currently administered by the subcutaneous route [79,80,81,82].

Respiratory vaccination via the aerosol route has been evaluated with two vaccine formulations, nebulized liquid and dry power vaccine (Table 1). Respiratory delivery of measles vaccine has been studied for 30 years and the WHO Measles Aerosol Project was established with the goal to license one respiratory mode of measles vaccine delivery. Understanding the average volume of inhaled vaccine delivered to the deep lung is important to determine the dosage received upon aerosolization. Studies suggest that an effective immune response is achieved when the vaccine reaches the deep lung and, therefore, aerosolized particles must be <5 µM in size [83]. Evaluation of liquid nebulization in the macaque animal model demonstrated that tidal volume, the lung volume representing total volume of air displaced during inhalation and expiration during normal breathing, is similar to that of children. The studies also showed that the effect of breathing parameters (i.e., tidal volume, breaths/min, and inspiratory to expiratory ratio) on dose inhalation differs with the various nebulizer devices [84]. Testing of a nebulizer device used extensively in Mexico to deliver liquid aerosolized vaccine demonstrated that approximately 30% of the aerosolized vaccine is of the <5 µM particle size needed to reach the deep lung, and that about 20%–30% of the dose is likely inhaled [84,85]. Aerosol delivery generates an immune response with no adverse reactions. The clinical trials reviewed below used liquid aerosolized measles vaccine which has been most extensively evaluated. The discussion below is limited to studies within the target age groups recommended for administration of MCV1 and a second dose of MCV (MCV2) using current measles vaccine formulations. In clinical trials, aerosol delivery boosted the immune response in school aged children, but initial results in infants were less promising. Several studies showed that infants under 12 months of age responded to vaccine but did not meet the strict non-inferiority threshold compared to subcutaneous delivery. However, increasing the exposure time of infants to the nebulized vaccine demonstrated comparable immunity to subcutaneous injection [83,85,86,87,88,89]. Infants vaccinated for an increased time duration (1–2.5 min of exposure instead of 30 s) by aerosol delivery of the vaccine demonstrated higher antibody responses compared to vaccination at the reduced exposure time [85,90]. When the time of aerosol vaccination was increased to 2.5 min, greater than 95% of infants seroconverted with protective levels (>120 mIU/mL) of antibody as measured by plaque reduction neutralization assay (Table 1) [85]. The use of aerosol vaccination as a primary immunization is less effective when given at the shorter 30 s exposure time, however, studies suggest that increasing the exposure time of aerosol delivery of a primary dose can increase the efficacy of this alternative route of immunization. Aerosol delivery is effective to provide a second dose of vaccine at the shorter 30 s exposure time, and MCV2 delivery via aerosol performed as well and often better than subcutaneous delivery as measured by the antibody response [86,88,91,92]. Measles vaccine formulations perform unequally when delivered by aerosol. The Edmonston-Zagreb strain appears to be the most immunogenic, withstanding aerosolization without loss of vaccine titer, and the immunogenicity as measured by antibody titer was comparable to subcutaneous injection. The MMR II^®^ vaccine (Merck, Kenilworth, NJ, USA) also may be delivered via aerosol with slightly higher antibody titers compared to subcutaneous injection in a study of school aged children, whereas the Schwarz strain is inactivated by the nebulizer [83,86,88]. This loss in potency is likely due to differences in the stabilizing agents and excipients used for the measles vaccine formulations.

Additional alternative vaccine delivery methods are newer in development and because of their additional benefits such as cold-chain elimination prospects, pre-clinical data is included for discussion. Measles vaccine is also being studied for aerosol delivery in a dry powder formulation (Table 1). The dry powder vaccine is reconstituted in vivo by moisture in the respiratory tract. The advantages to the dry powder formulation are that it has greater thermostability and is not dependent on maintenance of the cold chain [83,93]. In initial studies, the dry powder formulation did not perform as well as subcutaneous delivery of measles vaccine because the large particle size of the powder limited access of particles to the deep lung (Table 1) [93]. Improvements in the production of dry powder vaccine preparations by CO_2_ assisted nebulizer with bubble dryer (CAN-BD) produced a smaller particle size of 3–5 µM, the size required to reach the deep lung [83,93]. Subsequent studies with the improved preparation methods have evaluated two devices for the delivery of dry powder vaccines, the Puffhaler^®^ (Aktiv-Dry, Boulder, CO, USA) and the BD Solovent^®^ (Becton-Dickinson, Franklin Lakes, NJ, USA), both have been shown to be equally effective in delivering measles dry powder vaccines in a macaque model [83]. A study of delivery of dry powder MeV vaccine using both devices in macaques demonstrated an immune response as effective as subcutaneous delivery of MeV vaccine. The animals produced effective humoral and cellular immune responses that protected animals from challenge and were characterized by high avidity antibodies, the production of IgA in the respiratory mucosa and the activation of memory cells [94]. The dry powder vaccine formulation evaluated in phase 1 clinical trials was immunogenic and produced no adverse events in seropositive adult males [95].

One of the most promising alternative delivery technologies is the microneedle patch (Table 1). Microneedles have been under development since the mid-1990s and the most recent formulations used to evaluate measles vaccine delivery include dissolving microneedles. Dissolving microneedles can be formulated from inert material such as polymers and sugars including trehalose, sucrose, arginine and hepatagluconate in which vaccine is encapsulated into the tip of the needle. These materials allow more effective delivery upon patch application, increase the stability of the vaccine and are approved for human use [101,102]. The microneedles dissolve in the skin, releasing the vaccine and eliminating the need for sharps or biohazardous waste disposal [102]. An additional benefit to microneedle patch usage is that it may be administered by minimally trained individuals [100,102]. Studies in macaques have demonstrated an equivalent antibody and cellular response following vaccination with measles microneedle patches and subcutaneous vaccination (cellular response: Melissa Coughlin, unpublished data), with no significant reactivity at the site of patch placement [100]. This new delivery method will not be available in the near future because development of microneedle vaccines for measles and rubella is still in the preclinical phase. Candidate vaccines will need to be evaluated in clinical trials and successfully negotiate regulatory pathways. Though the microneedles will be fabricated with the live attenuated vaccines that are currently produced by vaccine manufacturers, a plan for large scale commercial production of microneedles under GMP conditions will be required.

## 7. Global Control and Prospects of Eradication

Measles is on a very short list of potentially eradicable diseases. Following smallpox eradication in the 1970s, the second human disease targeted for eradication is polio. One polio serotype, type 2, has been officially declared eradicated with type 3 also likely eradicated [103]. Many lessons learned during the polio eradication efforts have helped in the efforts to control measles [44]. Furthermore, the eradication of a related *Morbillivirus* of cattle, rinderpest virus, supports the biological feasibility of measles eradication [104]. MeV has no animal reservoir, a highly effective and safe vaccine is available, and a surveillance network has been established to identify and respond to cases and outbreaks. Molecular data demonstrate that chains of measles transmission are frequently interrupted by vaccination (Figure 2) [11,32].

In 2010, the World Health Assembly (WHA) identified goals for measles control that were to be achieved by 2015. These included coverage with MCV1 of 90% at the national level and 80% in all districts, reported measles incidence of less than 5/million population, and 95% measles mortality reduction compared to 2000 [105,106]. Though significant improvements have been made, with an estimated 20.3 million deaths prevented by measles vaccination in the last 15 years, global control efforts have lagged and the global community has not met the WHA goals [44]. As of 2014, estimated global coverage with MCV1 is 85%, and as of 2015, only 61% of countries have reached >90% MCV1 coverage. Coverage with MCV2 also increased significantly globally, with six countries (Angola, Malawi, Mozambique, Nepal, Sierra Leon, and Zimbabwe) adding a routine MCV2, bringing global MCV2 coverage to 61% [44,50,51]. In 2015 alone, SIAs vaccinated 184 million individuals, with most of these efforts also providing other childhood health interventions including vitamin A supplementation, rubella vaccination, and deworming interventions [44]. More than half of all unvaccinated children reside in six countries, the Democratic Republic of Congo, Ethiopia, India, Indonesia, Nigeria, and Pakistan [44,50].

Though the 79% decrease in global measles deaths between 2000 and 2015 fell short of the WHA goal of 95% reduction, the impact on mortality in children under the age of five has been significant [32,44,50]. With an estimated 23% of the decrease in mortality in children under the age of five from 1990 to 2008 accounted for by measles reduction, measles control efforts have significantly contributed to the fourth United Nations Millennium Development Goal, to reduce overall childhood mortality by two-thirds compared to 1990 [44,106].

Of course, many challenges remain before measles eradication can be considered. The greatest challenge is to increase vaccine coverage in all countries. This will require strengthening of the public health infrastructure [106]. The development of new strategies to deliver measles vaccines will help to improve coverage. In many regions, the cold-chain requirement of the measles vaccine is difficult to maintain and financially burdensome. Improved vaccine formulations that offer greater thermostability such as the microneedle patch may lead to improved coverage. Furthermore, the availability of medically trained personnel to administer subcutaneous vaccinations is limited in many countries; therefore, a vaccine delivery technique such as the microneedle patch that does not require medically trained personnel could contribute significantly to improving global MCV coverage. All three of the discussed novel vaccination strategies, aerosol, dry powder and microneedle patches have been estimated to reduce cost per dose due to improved thermostability, reduction of sharps waste disposal, reduced need for trained personnel, and reduced wastage of unusable vaccine [107,108]. The dry powder and microneedle vaccines have the additional advantage of being available in single dose packages which reduces wastage.

Vaccine hesitancy or exemption can lead to relatively large outbreaks even within regions with high vaccination coverage [109,110,111,112]. Many countries need to improve case-based surveillance for measles and rapid confirmation of cases because of the constant threat of measles importations [11,41]. Therefore, elimination efforts require not only the establishment of surveillance capacity but also the ongoing maintenance of laboratory capacity within the GMRLN [32]. Finally, a measles eradication goal will require a significant commitment of financial resources from both governmental and non-governmental organizations.

In 2010, an expert advisory panel stated that measles can and should be eradicated [32,105,106]. Measles has been verified as eliminated from the region of the Americas and the remaining five WHO regions have adopted measles elimination goals. Five out of the six regions have established regional elimination verification commissions (Figure 1) [44,51,52]. Sustained efforts to improve vaccine coverage and to maintain adequate surveillance will help to reduce measles incidence globally, allowing all children worldwide to avoid this previously inevitable childhood disease.

## Figures and Tables

**Table 1:** Summary of MeV Alternative Vaccination Studies Compared to Subcutaneous Injection ^a^.

Vaccine Formulation	Study Population	Immune Response ^b^	Vaccine Virus Strain	Comments	Study Year ^c^	Ref.
Aerosol	4–6 month old infants First dose	Seroconversion d (↓)	Edmonston-Zagreb (Institute of Immunology, Zagreb) Schwarz (Smith-Kline-RIT)	Administered aerosol exposure for 10 s. Aerosol dose given at 10× SQ f dose with an assumed 10% delivery. Less than 50% seroconversion in SQ.	1987	[96]
Aerosol	4–6 month old infants First dose	Seroconversion (~)	Edmonston-Zagreb (Institute of Immunology, Zagreb)	Longer exposure time higher Ab g. PRN titers lower than older infants in all groups.	1984	[90]
Aerosol	9 month old infants First dose	Seroconversion seroprotection e and T cell response (↓)	Edmonston-Zagreb (SII)	Lower dose administered in aerosol group.	2006	[97]
Aerosol	9 month old infants First dose	Seroconversion, seroprotection and T cell response (~)	Edmonston-Zagreb (SII)	Exposure time increased to 2.5 min. IFN-γ production equivalent.	2011	[85]
Aerosol	9–11.9 month old infants First dose	Seroprotection and seroconversion (↓)	Edmonston-Zagreb (SII)	Administered aerosol exposure 30 s. Difference of 9.4% did not meet non-inferiority (5%) criteria.	2015	[87]
Aerosol	12 month old infants First dose	Seroconversion, seroprotection and T cell response (↓)	Edmonston-Zagreb (Mexican National Institute of Virology)	Lower dose administered in aerosol group. All children boosted at 15 months SQ. IFN-γ production equivalent.	2004	[98]
Aerosol	5–6 year old children	Seroconversion and Seroprotection (↑)	Edmonston-Zagreb (Swiss Serum and Vaccine Institute)	Booster dose. MR used.	2002	[92]
Aerosol	6–7 year old children	Seroconversion and Seroprotection (↑)	Edmonston-Zagreb (SII) Attenuvax (Merk MMRII)	Aerosol delivery for 30 s sufficient for boosting response in school age children. MMR vaccine used.	2014	[86]
Aerosol	6–8 year old children	Seroconversion (↑)	Edmonston-Zagreb (Swiss Serum and Vaccine Institute)	Aerosol delivery for 30 s M and MR vaccines used. Better boosting of Ab response in aerosol groups at all titers even “low dose” 1000 pfu.	2002	[91]
Aerosol	5–14 year old children	Seroconversion (↑) with Edmonston-Zagreb Seroconversion (↓) with Schwarz	Schwarz Edmonston-Zagreb (SmithKline Beecham)	Administered aerosol exposure 30 s. Schwarz vaccine shown to lose potency following 2 min nebulization. Aerosol delivery boosted response in school aged children.	2000	[88]
Aerosol	2 year follow up previous study	Seroconversion Maintenance (↑) with Edmonston-Zagreb		86% of children from initial study included; 6% titers below seropositive cutoff compared to 13%–19% SQ.	2000	[99]
Aerosol	6 year follow up previous study	Ab titers and proportion seroprotected (↑)		70% of children from initial study included.	2007	[89]
Dry powder	Cynomolgus macaques	Ab response (↓) Upon challenge secondary response peaked earlier	Edmonston-Zagreb (Birmex or Berna)	Iron tracer study demonstrated most of dose did not reach deep lung.	2007	[93]
Dry powder	Rhesus macaques	Ab response (~) T cell response (↑) Protection from challenge Memory response	Edmonston-Zagreb (SII)	Puffhaler® and BD Solovent® administration comparable Smaller particle size dry powder.	2011	[94]
Dry powder	18–45 year old seropositive males	Ab response (~)	Edmonston-Zagreb (SII)	Administration by Puffhaler® and BD Solovent® devices was well-tolerated, no adverse events reported. Baseline titer high, seroconversion only detectable in 20%–30% individuals.	2014	[95]
Microneedle	Rhesus macaques	Ab titer (~)	Edmonston-Zagreb (SII)	Increased stabilization. No site specific reactions noted.	2015	[100]

^a^ Study selection limited to those using current vaccine strains and published in English. Studies of liquid aerosol restricted to target age groups; ^b^ All comparisons are made to subcutaneous (SQ) injection of the same vaccine; ^c^ Year of study publication; ^d^ Defined as a 4-fold rise in titer between pre and post vaccination samples; ^e^ Defined as titer of ≥120 mIU/mL; ^f^ Abbreviation for subcutaneous injection; ^g^ Abbreviation for antibody; Symbol after immune response description indicates higher (↑), equivalent (~), or lower (↓) response.
